# The Role of B-Cells and Antibodies against *Candida* Vaccine Antigens in Invasive Candidiasis

**DOI:** 10.3390/vaccines9101159

**Published:** 2021-10-10

**Authors:** Manisha Shukla, Pankaj Chandley, Soma Rohatgi

**Affiliations:** Department of Biosciences and Bioengineering, Indian Institute of Technology Roorkee, Roorkee 247667, UTT, India; mshukla@bt.iitr.ac.in (M.S.); pankaj_c@bt.iitr.ac.in (P.C.)

**Keywords:** systemic candidiasis, humoral immunity, B-cells, antibodies, vaccines

## Abstract

Systemic candidiasis is an invasive fungal infection caused by members of the genus *Candida.* The recent emergence of antifungal drug resistance and increased incidences of infections caused by non-albicans *Candida* species merit the need for developing immune therapies against *Candida* infections. Although the role of cellular immune responses in anti-*Candida* immunity is well established, less is known about the role of humoral immunity against systemic candidiasis. This review summarizes currently available information on humoral immune responses induced by several promising *Candida* vaccine candidates, which have been identified in the past few decades. The protective antibody and B-cell responses generated by polysaccharide antigens such as mannan, β-glucan, and laminarin, as well as protein antigens like agglutinin-like sequence gene (Als3), secreted aspartyl proteinase (Sap2), heat shock protein (Hsp90), hyphally-regulated protein (Hyr1), hyphal wall protein (Hwp1), enolase (Eno), phospholipase (PLB), pyruvate kinase (Pk), fructose bisphosphate aldolase (Fba1), superoxide dismutase gene (Sod5) and malate dehydrogenase (Mdh1), are outlined. As per studies reviewed, antibodies induced in response to leading *Candida* vaccine candidates contribute to protection against systemic candidiasis by utilizing a variety of mechanisms such as opsonization, complement fixation, neutralization, biofilm inhibition, direct candidacidal activity, etc. The contributions of B-cells in controlling fungal infections are also discussed. Promising results using anti-*Candida* monoclonal antibodies for passive antibody therapy reinforces the need for developing antibody-based therapeutics including anti-idiotypic antibodies, single-chain variable fragments, peptide mimotopes, and antibody-derived peptides. Future research involving combinatorial immunotherapies using humanized monoclonal antibodies along with antifungal drugs/cytokines may prove beneficial for treating invasive fungal infections.

## 1. Introduction

### 1.1. Invasive Candidiasis

Candidiasis broadly refers to fungal infections caused by members of the genus *Candida*. *Candida* species can exist inside human hosts as commensals and have emerged as important agents of opportunistic infections. Superficial infections are frequently observed in response to overgrowth or disruption of microbial flora, and/or environmental changes in individuals. However, in the event of breakdown of tissue barriers and during immune-compromising conditions, superficial infections lead to dissemination of *Candida* in the bloodstream, which is then referred to as invasive or systemic candidiasis [1]. Systemic candidiasis is one of the most common bloodstream infections in hospitalized patients worldwide [2]. According to the Centers for Disease Control and Prevention (CDC), the mortality attributed to systemic candidiasis is around 40%–70% worldwide, even with the use of antifungal therapies [3]. Globally, it is the fourth most common nosocomial bloodstream infection, which affects intensive care unit patients [4]. Every year, systemic candidiasis affects more than 250,000 people worldwide causing approximately 50,000 deaths [5]. More than 90% of the invasive infections are attributed to five *Candida* species, which include *C. albicans*, *C. tropicalis*, *C. glabrata,* *C. parapsilosis*, and *C. krusei*. More recently, a multidrug-resistant *Candida* species, *C. auris* has been linked to major outbreaks of invasive infections in healthcare facilities around the globe [6]. Currently, five classes of antifungal agents: polyenes (amphotericin B), azoles (fluconazole, itraconazole, posaconazole, voriconazole, and isavuconazole), echinocandins (caspofungin, micafungin and anidulafungin), allylamines (terbinafine), and antimetabolites (flucytosine) are used to treat invasive candidiasis [7]. Despite improvements in antifungal therapy, morbidity and mortality in patients with invasive candidiasis remain very high. Further, adverse side effects and toxicity of antifungal drugs limit the use of these drugs. In addition to this, the emergence of antifungal drug resistance in *Candida* species has been increasing over the past decade and multidrug-resistant mechanisms to *Candida* species pose a serious threat to public health worldwide. According to the CDC’s report regarding the antibiotic resistance threat in 2017, more than 34,000 cases and 1700 deaths annually were due to drug-resistant *Candida* species [8]. Additionally, the widespread distribution and emergence of new *Candida* strains is a major cause of concern. Apart from antifungal drug resistance, longitudinal studies have detected a shift towards non-albicans *Candida* (NAC) species for the past few decades [9]. Population-based studies suggest that the geographical distribution of *C. albicans* to NAC species varies by region [10].

Increased interest in the development of new vaccines against *Candida* infection is crucial for high-risk individuals such as immunocompromised patients, premature infants, cancer patients, and those with invasive treatments for long periods in hospital settings. To resolve the burden and challenges posed by *Candida* mediated systemic candidiasis, there is a strong specific medical need for vaccine/s or immunotherapies that target *Candida* species. Therefore, new alternative immunotherapeutic approaches are urgently needed to treat systemic candidiasis caused by *Candida* species. For a long time, the role of cellular immunity has overshadowed the contribution of humoral immunity in host defense against invasive candidiasis. The main aim of this review is to disseminate currently available information and experimental evidence regarding the role of B-cells and antibody-mediated immune responses against *Candida* vaccine antigens from studies done mostly in *C. albicans*. In this review article, we have summarized the lead anti-*Candida* vaccine candidates and humoral immune responses induced by them for conferring protection. Identification of fungal antigens, which elicit protective antibodies can initiate the design of multi-valent or multi-epitope *Candida* vaccine/s. Recent advances regarding monoclonal antibodies and their mechanisms of protection, anti-idiotypic antibodies, single-chain variable fragments, and peptide mimotopes are discussed, which may be useful for the development of direct antibody-based, as well as combination immunotherapies against invasive candidiasis.

### 1.2. Innate Immunity in Invasive Candidiasis

The physical barriers, like skin and the mucosal epithelial surfaces existing in the mouth, upper airways, the gastrointestinal and genitourinary tracts, are mainly considered the first line of defense against fungal pathogens [11]. Epithelial cells also play important roles during antifungal responses and both renal epithelial and endothelial cells have been implicated in conferring protection against systemic candidiasis [12]. The innate immune system not only provides an essential early response against fungal infections, but also stimulates several responses mediated by the adaptive immune system [13]. As the first step in the activation of innate immunity, host pattern recognition receptors (PRRs), such as integrins, lectins, and Toll-like receptors (TLRs) recognize pathogen-associated molecular patterns (PAMPs) present on *Candida*, and serve as efficient receptors for phagocytosis of opsonised *Candida* yeasts and induction of proinflammatory cytokines for recruiting and activating phagocytes [14]. The complement system, which is a part of innate immunity, also plays a central role in host defense against *Candida*, as demonstrated by various studies using complement protein C3 depleted or C3 deficient animals [15,16]. In addition to its ability to facilitate phagocytosis by opsonization, the activation of the complement system also generates anaphylatoxins (C3a, C4a, and C5a), which mediate several pleiotropic effects [17]. The role of phagocytic cells, such as macrophages, monocytes, dendritic cells (DCs), and neutrophils has been established in innate resistance to disseminated candidiasis. Macrophages can phagocytose and eliminate *Candida* yeast cells, thereby limiting the fungal burden early upon infection [18]. Macrophage depleted mice cleared *C. albicans* more slowly, showed significantly impaired survival and had significantly increased *Candida* CFUs (colony forming units) during disseminated candidiasis [14,19]. Monocytes encounter *Candida* early during infection and were found more effective in killing *C. albicans* than DCs or macrophages [20]. Monocyte deficient mice are more susceptible to infections with *C. albicans* [21], and monocytes exposed to *C. albicans* produce tumor necrosis factor-α (TNF-α), which is required for surviving systemic candidiasis [22]. Furthermore, both human blood classical and non-classical monocytes exhibit candidacidal activity [23]. DCs play a prominent role in host defense against invasive candidiasis, as they are capable of phagocytosis and antigen presentation, serving as a link between innate and cell-mediated antifungal immunity [24,25]. DCs discriminate between yeast and hyphae by producing opposing sets of cytokines (Th1/Th2) following phagocytosis [14]. Although DCs can ingest and kill *Candida*, they were reported to be less efficient than macrophages at fungal killing [20]. Neutrophils are of major importance in host defense against *Candida* infections, since neutrophil activation is essential for clearance of *Candida*, with neutropenia being a major risk factor for invasive fungal infections [23]. The role of neutrophils in mediating *Candida* killing has been established and they are the only immune cells capable of successfully inhibiting the conversion of *Candida* blastopores into hyphae, a key fungal virulence trait [12,26]. Neutrophils are indispensable for effective host defense during invasive candidiasis in mice [26]. Gardner et al. showed the importance of neutrophils in the protection against systemic candidiasis in neutropenic patients [27]. Uppuluri et al. reported antibody-mediated enhancement of phagocytosis and killing of *C. albicans* by neutrophils [28]. A recent study reported increased recruitment of neutrophils in kidneys of vaccinated mice and antibody-mediated neutrophil killing of *C. tropicalis* in disseminated candidiasis [29]. Natural killer (NK) cells play a crucial role in the early defense against murine systemic *C. albicans* infection [30]. It has been demonstrated that the absence of NK cells led to an increased susceptibility to both primary and secondary systemic *C. albicans* infections in T/B/NK-cell-deficient mice, compared with T/B-cell-deficient SCID (severe combined immunodeficiency) mice [30]. The role of NK cells in systemic *Candida* infection has been reported using human NK cells, which could mediate direct cytotoxic effects on the fungus [31]. It has been shown that NK cells can damage *Candida* directly via cell surface receptors or indirectly via cytokines and interferons [32].

### 1.3. Adaptive Immunity in Invasive Candidiasis: Cellular Responses

In addition to innate immunity, adaptive immunity is essential for a successful elimination and development of optimal protective immunity against invasive candidiasis. Cell-mediated adaptive immune responses constitute the mainstay of host defense mechanisms against systemic candidiasis, wherein both CD4^+^ T helper cells and CD8^+^ cytotoxic T-cells are the predominant players involved in controlling *Candida* infection [13].

CD4 T-cells: Historically, CD4^+^ T-cell immune responses have been believed to be protective against *Candida* infection [33]. CD4^+^ T-cells are known to contribute towards Th1, Th2, and Th17 immunity during antifungal immune response [34]. Although CD4^+^ T-cells do not have direct cytolytic activity, they still play a critical role against *Candida* infection. The significance of the CD4^+^ T-cell response in inducing protective immunity against *C. albicans* is indicated by the prevalence of oropharyngeal candidiasis in AIDS (acquired immunodeficiency syndrome) patients whose CD4^+^ T-cell count is depleted [35]. It has been demonstrated that protection against systemic candidiasis is associated with both Th1 and Th17 responses [36,37,38,39]. Furthermore, protective vaccine responses are associated with robust Th1 and Th17 responses [40,41].

CD8 T-cells: CD8^+^ T-cells are known to mediate resistance to systemic fungal infections, primarily through their direct cytotoxic activity and cytokine secretion [42,43]. The role of CD8^+^ T-cells in the direct killing of fungi has been established [44] and cytokine secretion (mainly interferon gamma [IFN-γ] and TNF-α) has been demonstrated as one of the main effector mechanisms through which CD8^+^ T-cells can restrict fungal infection [45].

Cytokines: Multiple different cytokines and chemokines are associated with protection against *Candida* infection. Studies have shown that certain cytokines enhance phagocyte killing of *Candida* species and specific cytokines expressed by antigen presenting cells like DCs and macrophages are crucial for the differentiation of T-helper (Th) cells [46,47]. Th1 cells secreted cytokines can also activate B-cells, resulting in the secretion of antigen-specific antibodies against *Candida*. Protection against fungal infections has been extensively correlated with Th1/Th17 mediated cellular immunity [48,49]. The role of granulocyte-macrophage colony-stimulating factor (GM-CSF), generally recognized as a proinflammatory cytokine, has been demonstrated in the augmentation of neutrophil mediated killing of *C. albicans* and a decrease in mortality was observed in mice treated with recombinant GM-CSF during disseminated candidiasis [49]. The protective role of IFN-γ against disseminated candidiasis has been established using mice deficient in IFN-γ and IFN-γ receptor, which were highly susceptible to candidiasis [50,51]. IFN-γ has been shown to enhance neutrophil mediated damage of *Candida* strains [10,52], and a study by Londono et al. implicated the role of IFN-γ during the development of *Candida*-associated abscesses in a mucosal model [53]. While administration of IFN-γ exacerbated *Candida* infection in mice, neutralization of endogenously synthesized IFN-γ by a specific antibody has been shown to prevent the development of a protective Th1 response [54]. Further, recombinant IFN-γ therapy has been shown to improve an immunological response in patients with systematic candidiasis [55]. In addition, mice deficient in IL-18 (which plays a crucial role in the induction of IFN-γ) were found more susceptible to disseminated candidiasis [34,56]. IFN-γ can also contribute to anti-*Candida* host defense by inducing nitric oxide production by macrophages, as well as *Candida*-specific immunoglobulin production [34,57]. The role of TNF-α in the development of protective Th1 immune response has been demonstrated against *Candida* infection [58], and depletion of TNF-α by etanercept treatment rendered mice more susceptible to disseminated *C. tropicalis* infection compared to controls [59]. TNF-α and interleukin IL-1 mediated activation of macrophage and neutrophils is critical during disseminated candidiasis. IL-1 cytokine has been seen effective against disseminated candidiasis and using knockout mice, Vonk et al. showed that both IL-1α and IL-1β are required for the induction of protective Th1 responses against disseminated candidiasis [60]. The presence of an increased concentration of IL-2 in mice spleen post-infection provided protection during systemic *C. albicans* infection [61] and Beno et al. demonstrated that IL-2 activated lymphocytes can limit the growth of *C. albicans* hyphae [62]. Circulating concentrations of IL-6 cytokine have a prognostic value for the outcome of sepsis patients and previously published studies showed increased susceptibility to systemic candidiasis in IL-6^−/−^ deficient mice [63,64]. It has been shown that IL-7 improves survival in fungal sepsis, wherein IL-7 was found to enhance the activation and proliferation of lymphocytes and also enhanced the production of INF-γ suggesting its role as an immunotherapeutic agent [65]. The role of IL-12 cytokine in healing infections against murine candidiasis is known [66] and IL-12 production has been shown to correlate with induction of Th1 phenotype in murine candidiasis [67]. Netea et al. reported the differential role of IL-18 in host defense against disseminated *C. albicans* infection [56] and IL-18 has been shown to play a protective role against disseminated *C. albicans* infection [68]. A previous report demonstrated that IL-23 contributes to antifungal defense by promoting neutrophil activity via an NK cell- and GM-CSF-dependent pathway [69]. Recently, a study reported that the absence of IL-23 led to a rapid loss of tissue-infiltrating neutrophils and monocytes as well as tissue-resident macrophages and dendritic cells in mice kidney during systemic candidiasis [70]. Th17 cells have been shown to play a major role in anti-*Candida* immunity at mucosal surfaces. It has been shown that IL-17 secreted by Th17 cells keeps fungal infection in check by recruiting neutrophils and inducing defensin secretion by epithelial cells at mucosal sites [36]. In a recent study, IL-17A has been shown to confer protection against invasive candidiasis [71], and IL-17 deficiency has been shown to enhance susceptibility to *C. albicans* infections at mucosal sites [72]. Additionally, various cytokines, such as IL-1, IL-6 and transforming growth factor (TGF-β), are involved in the development, proliferation and activation of Th17 cells [73]. Unlike Th1 cytokines, Th2 cytokines may antagonize effective immunity against systemic candidiasis. Th2 immune response is associated with the increased fungal burden and disease exacerbation [50,74]. However, a requirement of IL-4 has been shown for the development of protective immunity against systemic *Candida* infection [75] and a previously published study has shown that the presence of TGF-β may be required for optimal development of protective Th1 responses against systemic candidiasis [76]. Further, an immunoregulatory role has been speculated for IL-4, IL-10, IL-13, and IL-33 during *C. albicans* mediated systemic infection.

### 1.4. Adaptive Immunity in Invasive Candidiasis: Humoral Responses

Although cellular immunity has been considered to be the central component during systemic candidiasis, there is an important role of humoral immunity in controlling invasive fungal infections. Although the complement system, collectins, and antimicrobial peptides are part of humoral immunity, the primary focus of this section is to evaluate the humoral responses mediated by B-cells and antibodies that confer protection during systemic candidiasis.

#### 1.4.1. B-Cell Responses in Invasive Candidiasis:

In a previously published study, Sinha et al. showed that B-cell deficiency did not increase the susceptibility of the animals to *C. albicans* mediated candidiasis [77]. Further, a previous study showed that B-cell deficiency had no relation with the increased susceptibility to *Candida* infection in mice [78]. In addition, a study by Bistoni et al. showed B-cells did not play a crucial role in protection against the *C. albicans* strain [79]. Previously published studies with SCID mice showed that the B-cell deficiency did not increase susceptibility to *C. albicans* infection [80,81]. However, in a study using germ-free B-cell knockout mice, it has been shown that these mice are susceptible to experimental systemic candidiasis, but resistant to mucosal and systemic candidiasis [82]. Jones-Carson et al. demonstrated that the thymic and extrathymic T-cells participate in mucosal immunity to *C. albicans* in the absence of B-cells, contributing to protective immunity to systemic candidiasis [18]. It has been shown that animals receiving vaginal CD3^−^CD5^+^ B-cells transferred from immune rats had lesser *Candida* CFUs when compared to controls, but showed a significant delay in fungal clearance when compared to animals administered with immune T-cells [83]. Another study by De Bernardis et al. showed that passive transfer of vaginal B-cells from *Candida*-immunized rats in naïve animals resulted in protection against vaginal candidiasis [84]. Further, *in vitro* addition of rituximab in PBMCs (peripheral blood mononuclear cells) to deplete B-cells, led to reduced anti-*Candida* responses [85]. Lilly et al. showed that mice deficient in T and B-cells (Rag-1 knockouts) survived both initial *C. dubliniensis*/*S. aureus* challenge and *C. albicans*/*S. aureus* rechallenge, and protection against lethal *C. albicans*/*S. aureus* was not mediated by adaptive immunity [86]. Several previous studies have implicated B1 B-cells and B1-derived natural IgM in anti-fungal immunity [87,88]. A study found that *C. albicans* was cleared more efficiently in TgVH3B4 mice after fungal inoculation than control mice [89]. Notably, *C. albicans* infection led to enhanced proliferation of B-1 cells, which resulted in increased numbers of B-1a B-cells and *C. albicans*-specific B-cells in TgVH3B4 mice, which may have a role in fungal clearance [89]. A recent study has demonstrated that on cloning antibody genes from B-cell cultures derived from patients infected with *C. albicans*, the generated antibodies were capable of stimulating opsonophagocytic macrophage activity *in vitro* and provided protection against disseminated candidiasis *in vivo* [90]. Notably, an antibody-independent function involving cytokine secretion has been identified in human B- cells, which can contribute to antifungal immunity [91].

#### 1.4.2. Antibody Responses in Invasive Candidiasis

**Figure 1:**
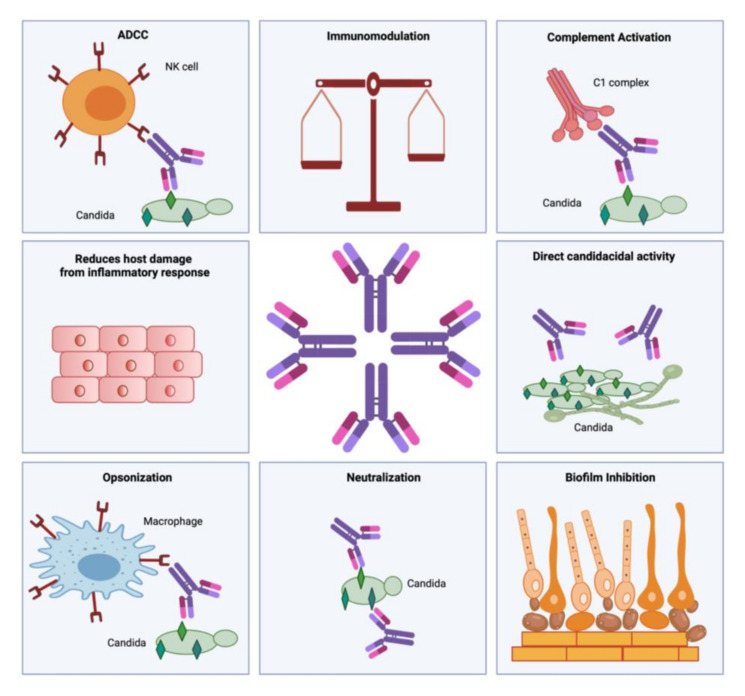
Schematic representation of antibody effector functions during invasive candidiasis. Antibody functions include neutralization, opsonization, complement activation, antibody dependent cellular cytotoxicity (ADCC), inhibition of biofilm formation, direct anti-candidacidal activity, immunomodulation and reduction of inflammatory damage. (Created with BioRender.com).

Antibodies are the effector molecules of the adaptive immune responses, which restrict the fungal burden and aid in its clearance [50]. Effector immune responses of antibodies include neutralization of toxins, prevention of pathogen adherence to host cells, opsonization, and antibody-dependent cellular cytotoxicity (ADCC) [92]. Other anti-fungal antibody immune response includes inhibition of biofilm formation, complement activation, phagocytosis, inhibition of germ tube formation, immune modulation, and fungal growth inhibition [50] (Figure 1).

Antibody isotypes IgM, IgG, and IgA are the main antibodies produced against fungal pathogens. IgA, which is a predominant antibody involved in conferring immunity at mucosal surfaces, is known to prevent the binding or attachment of *C. albicans* to human oral epithelial cells [93]. Maiti et al. found that B-cell deficient mice were unable to generate anti-*Candida* antibody response, while controls with functional B-cells were able to generate protective antibody response against *Candida* infection. The B-cell deficient mice were more prone to death, had reduced body weight, and were also found to be more susceptible to *Candida* infection. This finding suggested that B-cells and antibodies are responsible for the protection against *Candida* infection [94]. The role of anti-*Candida* antibodies in passive immunization and protection of the host against candidiasis has been studied by Cassone et al. [95]. However, the function of vaginal antibodies in the pathogenesis of vaginal candidiasis remains unclear [96]. A previously-published study found that vaginal anti-*Candida* IgA antibody was not protective against recurrent vaginal *C. albicans* infection, and the vaginal anti-*Candida* IgA and IgG antibodies levels were similar in women with or without vaginal candidiasis [97]. On characterizing anti-*Candida* IgA, IgE, IgG, and subclass (IgG1, IgG4) antibody levels in serum and vaginal washes from women with or without vulvovaginal candidiasis, another study demonstrated significantly higher levels of IgA in vaginal washes and lower in serum, present in patients. Additionally, similar levels of serum anti-*Candida* IgA and IgG antibodies were observed in women with or without a history of *Candida* vaginitis [98]. A study by Bernardis et al. reported that the anti-*Candida* human domain antibodies inhibited attachment of fungus to the vaginal epithelium, which protected rats from experimental vaginitis [99]. An earlier report demonstrated that antibodies generated against certain cell surface antigens of *C. albicans* protected mice against disseminated candidiasis [100]. Production of specific antibodies against fungal cell wall antigens like polysaccharides, glycoproteins, and enzymes confer protection against fungal infection in the host [101]. Vaccinated mice with a liposomal antigen delivery system containing *C. albicans* cell wall surface proteins demonstrated significantly higher serum levels of *C. albicans*-specific antibodies [102]. Moreover, antibody-based vaccines have been shown to confer protective immunity against systemic candidiasis [103].

## 2. Vaccine Candidates in Invasive Candidiasis

**Figure 2:**
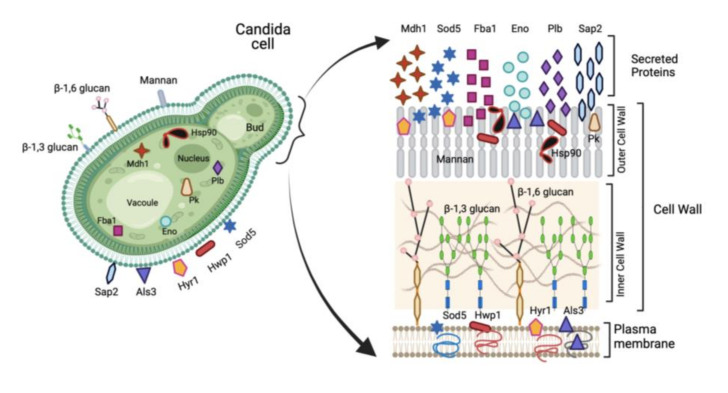
Schematic representation of *Candida* vaccine antigens. Yeast form of *Candida* depicted on left indicating major virulence antigens explored as vaccine candidates. Cytoplasmic location of Hsp90, Pk, Eno, Plb, Fba1 and Mdh1 is indicated along with various cell surface antigens (Sap2, Als3, Hyr1, Hwp1, Sod5, glucan and mannan). Cell surface is expanded on right to indicate secreted (Mdh1, Sod5, Fba1, Eno, Plb and Sap2), and cell wall associated antigens (Hyr1, Hwp1, Sap2, Als3, Eno, Hsp90, Pk, Plb, Sod5, Fba1, glucan and mannan). (Created with BioRender.com).

Rising incidence and mortality associated with systemic candidiasis, especially in high-risk groups such as immunocompromised individuals, elderly, newborns, cancer patients, and those with invasive treatments for long periods in hospital settings, necessitates the development of anti-*Candida* vaccine/s. Although several different anti-*Candida* vaccine candidates have been identified, only a few of them have progressed to clinical trial evaluation to date [104]. Various studies have evaluated the vaccine immunogenicity and efficacy against candidiasis mostly in animal models. However, many challenges still exist, which prevent clinical development of anti-*Candida* vaccine/s that can efficiently immunize subjects at risk of developing invasive fungal infections [105]. An effective fungal vaccine should be able to generate protective immune responses and immunological memory, which could provide protection against recurrent fungal infections. Several vaccines candidates have been studied to date that utilize live attenuated strains, fungal cell wall polysaccharides, recombinant proteins and/or glycoconjugates, as strategies for anti-*Candida* vaccines [104]. Likewise, many different strategies to enhance the activity of the vaccines have been published, including adjuvants and delivery systems [13]. Although many virulence antigens from *C. albicans* have been explored as vaccine candidates (Figure 2), only a few studies have been done to evaluate the contribution of humoral immunity in the protective immune potential conferred by them against invasive candidiasis experimentally.

As such, not much is known about the protective role of B-cell or antibody mediated responses during *Candida* mediated invasive candidiasis. In this review, we have summarized the lead anti-*Candida* vaccine candidates and humoral immune responses induced by them for conferring protection (Table 1).

### 2.1. Humoral Immune Responses to Mannan Polysaccharide

Approximately, 80% of the fungal cell wall is composed of polysaccharides, the main constituents of which are β-glucan, mannan, and chitin [106]. Mannan is mostly found as large N-linked polymers containing several hundred mannose residues associated with high-molecular-weight mannoprotein species. Being a leading polysaccharide antigen present on the cell wall of *C. albicans*, mannan is considered to be the main antigen for humoral immune responses. Detection of soluble mannan in serum is important for the diagnosis of invasive candidiasis [107]. When the mannan fraction was encapsulated into liposomes and used for mice vaccination, antibodies specific for the mannan fraction correlated with increased resistance to disseminated candidiasis, and both polyclonal and monoclonal anti-mannan antibodies were found to confer protection against disseminated candidiasis [100]. Detailed analysis of anti-mannan IgM monoclonal antibodies (B6 and B6.1) revealed the β-(1-2)-linked mannotriose epitope of mannan to yield protective antibodies [108]. The mechanism of protection by the anti-mannan monoclonal antibody (B6.1) was identified to be ingestion and killing of yeast cells by neutrophils in the presence of serum complement [109]. Cassone et al. were the first to report that acquired anti-*Candida* protection in a rat vaginitis model was mediated by anti-mannan antibodies, which were capable of transferring anti-*Candida* protection to naive, nonimmunized rats [110]. The protective anti-mannan antibody response was found to be T-cell-dependent in a separate study [111]. Interestingly, Mangeney et al. earlier demonstrated that *in vitro* anti-mannan antibody production is T-cell-independent-type 2, needing T-cell-derived cytokines, and mannan antigen can directly activate human B-cells to produce anti-mannan antibodies, independent of direct T and B-cell interactions [112]. Han et al. further reported that both the antibody response generated against the mannan vaccine or administration of anti-mannan antibodies could protect against a *Candida* infection in a mice model of vaginal candidiasis [113]. Zhang et al. found that anti-mannan IgG antibodies could initiate a classical pathway by C3 deposition on *C. albicans*, enabling complement activation against disseminated candidiasis [114]. Further, they reported the ability of anti-mannan IgG antibody in the activation of both classical and alternate pathways of the complement system [114,115]. On investigating the human humoral responses against β-1,2- and α-linked oligomannoside epitopes present on mannan and mannoproteins, the anti-mannan IgG antibody response was found to be associated with commensal to the pathogenic transition of *C. albicans* [116]. Han et al. demonstrated that vaccination with mannan-BSA (bovine serum albumin) protein conjugate in mice conferred protection against disseminated and vaginal candidiasis by inducing protective antibodies [117]. On testing monoclonal antibody C3.1 (IgG3) obtained from mice immunized with a liposome-mannan vaccine, the protective potential of anti-mannan antibodies was observed to be dependent on epitope specificity, serum titer, and ability to rapidly and efficiently fix complement to the fungal surface, enabling enhanced phagocytosis and killing of the fungus [118,119,120]. The protective mannan monoclonal antibody C3.1 has been reverse engineered to develop a conjugate vaccine (having mannan disaccharide epitopes and chicken serum albumin) against *C. albicans*. This vaccine produced antibodies in rabbits that recognized the native cell wall phosphomannan and reduced fungal burdens in immunized rabbits when challenged with live *C. albicans* [121].

Bernardis et al. reported the presence of mannan-specific protective antibodies in vaginal fluids in rat vaginitis model of candidiasis, along with increased numbers of CD5^+^ B-cells, which show restricted VH usage and exert a primary role as first-line antibody producers against microbial cell surface antigens [122]. In addition, when a mannan-conjugate vaccine was prepared using human serum albumin as a carrier protein and administered in rabbits, it induced protective IgG mediated anti-*Candida* response [123]. Kozel et al. found that anti-mannan antibodies found in sera of normal donors show biological activities, which include complement activation and induction of opsonophagocytic killing of *C. albicans* [124]. Zhang et al. reported that a human anti-mannan monoclonal antibody (M1g1) plays a protective role in host resistance to systemic candidiasis by promoting the phagocytic killing of *C. albicans* yeast cells by mouse peritoneal macrophages and was required for activation of the mouse complement cascade [125]. A separate study found that this human anti-mannan antibody had a distinct Fc-independent effector function in the regulation of C3 deposition to *C. albicans* through the alternative pathway [126]. Nishiya et al. demonstrated that the role of human anti-mannan antibody (M1g1) in host resistance to systemic candidiasis is influenced by its IgG subclass [127]. On conjugating mannan with tetanus toxoid or BSA, high titers of mannan antibodies were found in rabbits but low titers of antibodies were observed in mice after immunization with both glyco-conjugate vaccines [128]. Replacing the glycosidic oxygen atom with a sulphur atom was found to increase the stability and immunogenicity of these two glyco-conjugate vaccines [129]. When mannan from *C. dubliniensis* was conjugated with human serum albumin and evaluated in the rabbit model, Paulovicova et al. found high levels of IgG and IgA antibodies, activation of B-lymphocytes, granulocytes, T-cells, and Th-1 cytokine production against *Candida*; indicating generation of antigen-specific humoral response as well as the induction of cellular immunity [130]. On administering a *C. albicans* mannan-human serum albumin conjugate vaccine in rabbits, the antiserum obtained effectively decreased the viability of *Candida* cells and inhibited fungal growth [131]. Xin et al. prepared synthetic glycopeptide vaccines by combining β-mannan with various *Candida* cell wall proteins. Using an antigen-pulsed dendritic cell-based approach for mice vaccinations, they found that these synthetic glycopeptides elicited specific antibody against both epitopes, thereby inducing protective immunity in mice against candidiasis [132]. In a separate study, Xin et al. found that among these six glycopeptide conjugates, Fba peptide bound to the β-1,2-mannotriose elicited the strongest protective response [103]. Furthermore, Xin et al. showed that addition of tetanus toxoid to the glycopeptide conjugate (β-1,2–mannotriose-Fba) results in a self-adjuvanting vaccine that promotes robust antibody responses without the need for additional adjuvant [133].

Notably, the mannan-derived α-oligomannoside vaccine conjugated with BSA also showed a protective role against *Candida* infection in rabbits. The sera obtained post-immunization exhibited a significant level of IgG and IgM anti-*Candida* antibodies and induced candidacidal activity of peripheral blood leukocytes [134]. Lipinski et al. synthesized a glycopolymer vaccine by conjugating polyacrylamide and chicken serum albumin with β-mannan trisaccharide epitope of *C. albicans*. High titers of IgG antibodies were seen after administration of the vaccine in the mice model [135]. Using a synthetic β-mannan trisaccharide epitope conjugated to a tetanus toxoid in a neutropenic rabbit model of *C. albicans* infection, Lipinski et al. demonstrated that antibody-mediated immunity plays a substantial role in combating *C. albicans* infections [136]. A heptamannoside mannan-BSA conjugate vaccine (M7-BSA) was evaluated in BALB/c mice and shown to induce enhanced antibody mediated and T-cell mediated response against *Candida* [137]. Han et al. compared a liposome-encapsulated mannan (Lipo-mann) vaccine with mannan-BSA conjugate (Conju-mann) vaccine in C5-deficient mice model and reported that mannan-BSA conjugate vaccine conferred protection even in the absence of C5, rendering it superior to Lipo-mann [138]. On comparing immune responses to pentamannoside (M5-BSA) and hexamannoside (M6-BSA) conjugates (bearing synthetic a-1,6-branched oligomannosides), M6-BSA conjugate was seen to induce a higher level of IgG antibodies than M5-BSA and enhanced candidacidal activity of polymorphonuclear cells, indicating a role of structure-based immunogenicity in the generation of protective immunity against a *Candida* infection [139]. On analyzing the cellular immune cell responses induced by M6-BSA and M5-BSA conjugates in mice, Paulovicova et al. found that both the conjugates activated CD4^+^ T-cells and neutrophils. The cytokine profile of immunized mice indicated upregulation of Th2 cell-mediated immune response induced by immunization with M5-BSA conjugate and upregulation of Th1 cell-mediated immune response induced by M6-BSA conjugate immunization [140]. Antisera elicited by immunizing rabbits with the *C. albicans* mannan-human serum albumin conjugate vaccine was able to inhibit the growth of different *Candida* species and IgG antibodies were found to be associated with protective anti-fungal immunity [141]. When glycopeptides consisting of β-1,2-mannan-peptide conjugates consisting of β-1,2-mannan and N-terminal peptide epitopes of *C. albicans* cell wall phosphomannan complex and recombinant Als1p protein were synthesized and conjugated with carrier proteins, they induced high levels of IgG antibodies and exhibited a self-adjuvanting property [142]. Mannan obtained from *C. glabrata* was able to induce splenocyte proliferation and also increased TNF-α and IL-4 cytokine levels. Additionally, mannan could modulate the activation of DCs as well as their antigen presentation activity, thereby influencing T-cell phenotype in response to stimulation [143]. Sendid et al. used synthetic oligomannosides and performed epitope mapping of monoclonal antibodies specific to *C. albicans* mannan. On analyzing the anti-mannan antibody response in the sera of patients infected with invasive candidiasis, unique specificities of β-1,2 mannotriose protective epitopes were identified [144].

### 2.2. Humoral Immune Responses to β-Glucan Polysaccharide

The β-1,3-glucans are structurally complex homopolymers of glucose, present on the *Candida* cell surface, which act as pathogen-associated molecular patterns. As a predominant extracellular polysaccharide antigen, β-glucans play significant roles in the induction of host protective immune responses and are a promising vaccine candidate against systemic candidiasis. Although β-glucan is poorly immunogenic, combined with compounds, such as diphtheria toxoid, results in a vaccine effective against both invasive and mucosal candidiasis. Curdlan (natural, linear β-(1,3) glucan without any β-(1,6) branching from *Alcaligenes faecalis*), zymosan (β-(1,3) glucan from *Saccharomyces cerevisiae*) and pustulan (a linear β-(1,6) linked β-glucan from lichen *Lasallia pustulata*), are experimental alternatives for β-glucan that are used for studying antifungal immune responses.

Bromuro et al. reported that mice immunized with *C. albicans* cells showed protective immune response by producing anti-β-glucan antibodies, which were protective in a mice model of disseminated candidiasis [145]. When mice were immunized with a glycoconjugate vaccine composed of β-glucan (laminarin) and diptheria toxoid (CRM197), the vaccine conferred protection against both systemic and mucosal candidiasis. The anti-β-glucan antibodies generated (IgG) were found to be protective and exhibited direct anti-fungal properties. In addition, a β-glucan monoclonal antibody (2G8), was found to inhibit *Candida* growth [146]. In a separate study, Rachini et al. showed that this monoclonal antibody could also confer cross protection in *Cryptococcus neoformans*, by exerting anticryptococcal activities *in vitro* and *in vivo* through its binding to *C. neoformans* cell wall β-glucan [147]. Further analysis of 2G8 (IgG2b) and IE12 (IgM) monoclonal antibodies revealed that protection by anti-β-glucan antibodies was associated with restricted binding specificity to β-1,3 glucan epitopes and inhibition of fungal growth and adherence, and the isotype of anti-β-glucan antibodies may affect details of the β-glucan epitopes recognized by these antibodies [148,149]. Recently, chimeric human-murine monoclonal antibodies derived from anti-β-glucan 2G8 mAb were expressed in plants and found to be protective in both systemic and mucosal models of candidiasis [150]. The β-glucan-CRM197 conjugate vaccine (Laminarin-CRM) administered in mice using MF59 adjuvant was also observed to confer protection against vaginal candidiasis, which was found associated with production of serum and vaginal anti-β-glucan IgG antibodies [151]. Further, the Laminarin-CRM conjugate vaccine, when administered with MF59, also protected mice against systemic *C. albicans* infection [152]. On evaluating glyco-conjugate vaccines using curdlan or synthetic β-glucan oligosaccharides, anti-β-(1,3)-glucan IgG antibodies were found to be protective and conferred protection to mice lethally challenged with *C. albicans*, compared to non-protective anti-β-(1,6)-glucan antibodies [152].

Anti-β-glucan antibodies are detectable in normal animal and human sera, and when elicited by glucan-based vaccines they can exert a fungicidal protective activity [106]. Studies have found that polyclonal, monoclonal and recombinant anti-β-glucan-like antibodies and peptide mimotopes are able to exert an *in vitro* and/or *in vivo* microbicidal activity against *C. albicans* [153]. Further, β-glucan antibodies, having a high reactivity to solubilized *C. albicans* cell wall β-glucan were identified in various animal serum samples and were seen to participate in immune response to pathogenic fungi [154]. Chiani et al. reported that naturally occurring human IgGs and, in particular, the IgG2 subclass, mostly recognize β-(1,6)-linked β-glucans of pustulan, compared to β-(1,3)-linked β-glucans of laminarin. Since IgG2 shows poor binding to Fc receptors on macrophages and neutrophils and does not efficiently fix complement, it may explain the low protective valence of the natural repertoire of anti-β-glucan antibodies in humans [155]. On analyzing anti-β-glucan antibodies profiles of candidemic and non-candidemic patients, candidemia patients were found to exhibit low levels of antibodies against β-(1,3)-glucan and high levels of antibodies to β-(1,6)-glucan. Interestingly, a significant correlation was observed between survival and level of mannoprotein MP65 antibodies [156]. Recently, a bispecific monoclonal antibody constructed by combining a mAb directed against 1,3-β-D-glucan, with a mAb recognizing MP65 mannoprotein has been used successfully for diagnosing invasive *Candida* infections [157]. Of note, a peptide derived from a single chain anti-idiotypic antibody against a yeast killer toxin was seen to protect BALB/c and SCID mice against systemic candidiasis. The peptide exerted strong candidacidal activity *in vitro* and since its activity could be neutralized by laminarin (β-1,3 glucan) and not pustulan (β-1,6 glucan), it was speculated that it interacted with β-glucan containing yeast killer toxin receptor present on *C. albicans* [158,159]. Additionally, Selvakumar et al. found that a single-chain anti-idiotypic antibody against a yeast killer toxin, inhibited the activity of β-1,3-glucan synthase, resulting in a strong cytocidal effect on the growth of *Candida* species [160]. Further analysis identified specific peptides in the anti-idiotypic antibody, which could interact with *C. albicans* β-glucan, and are responsible for exerting an inhibitory effect on the growth of *C. albicans* [161].

Huang et al. found that immunization with β-glucan particles complexed with ovalbumin, not only induced robust Th1- and Th17-based CD4 T-cell responses, but also induced strong humoral antibody responses (IgG2c) in mice [162]. Using larvae of *Galleria mellonella* as a model for *Candida* infection, a dose-dependent protection was observed when β-glucan was injected in high amount in the hemocoel of insects [163]. Inoculation of larval hemocytes with β-glucan increased the numbers of hemocytes and enhanced their candidacidal activity [164]. By incorporating β-glucan to a mannan tetanus toxoid conjugate, Lipinski et al. reported enhanced immunogenicity of this tricomponent vaccine through dendritic cells targeting via Dectin-1 [165]. Furthermore, Bundle et al. found that on incorporating a β-glucan dendritic cell ligand to a *C. albicans* B-cell β-1,2-mannotriose epitope attached to the Fba peptide T-cell epitope, the resulting three component synthetic vaccine could induce protective antibodies to all three epitopes of the fully synthetic construct [166]. Synthesized β-glucan- KLH (keyhole limpet hemocyanin) glyco-conjugates consisting of hexa-, octa-, deca- and dodecasaccharides elicited high titers of antigen-specific total and IgG antibodies in mice, suggesting the induction of functional T-cell-mediated and humoral immunity. Additionally, the size of β-glucan chains in glycoconjugate vaccine was seen to play a crucial role in its immunogenicity [167]. Further, Liao et al. reported that synthetic 6-O-branched oligo-β-glucan-based antifungal glycoconjugates elicited strong IgG antibody responses in mice and induced effective protection *in vivo* against systemic *C. albicans* infection via protective antibody generation [168]. Active immunization of mice with a synthetic linear β-nonaglucoside-BSA conjugate (structurally related to *Candida* β-(1,3)-glucan), was observed to induce an effective humoral immune response (IgG, IgM, IgA), and post-vaccination serum exhibited anti-*Candida* growth activity *in vitro* in a mucocutaneous infection model [169]. Mouse monoclonal antibodies (3G11 and 5H5) synthesized against synthetic nona-β-(1,3)-D-glucoside conjugated with BSA, interacted with *Candida* and demonstrated synergy with the antifungal fluconazole in killing *C. albicans in vitro*. Further, they also showed protective activity *in vivo*, suggesting their use in combinatorial antifungal therapy [170]. More recently, a *C. albicans* mutant with β-(1,3)-glucan exposed on its cell-surface was observed to induce the generation of protective antibodies against invasive candidiasis in mice, which was primed by IL-18 secretion [171]. On evaluating immune responses to curdlan, Kumar et al. found that B-cells were directly activated in response to curdlan, and this activation required NLRP3 (member of NOD-like receptor family) signaling for IgM antibody responses, implicating NLRP3 inflammasome in regulating β-glucan-induced anti-*Candida* innate and humoral adaptive immune responses [172]. It has been shown that Dectin-1, a TLR present on B-cells, specifically interacts with β-glucan present on the *Candida* cell wall. Seo et al. demonstrated that during fungal infection, β-glucan-stimulated Dectin-1 may cooperate with TLR4 to specifically enhance IgG1 production by mouse B-cells, suggesting a role of β-glucan in the B-cell mediated antibody response [173]. Hoogeboom et al. reported that human B-cells having BCRs with a heavy chain encoded by the IGHV3-7 gene family and a light chain encoded by the IGKV2-24 gene family, demonstrated high specificity for β-(1,6)-glucan, a major antigenic determinant of yeasts and filamentous fungi [174].

### 2.3. Humoral Immune Responses to Laminarin

Laminarin is a β-glucan entity isolated from brown algae, *Laminaria digitata*, consisting of β-(1,3) glucan repeating units, with sporadic β-(1,6) branches [175]. It is a non-fungal source of β-glucan, and a protective role of laminarin during systemic candidiasis infection has been reported by multiple studies. Polonelli et al. were the first to report a therapeutic activity of an anti-idiotypic antibody peptide fragment against yeast killer toxin during experimental systemic candidiasis. The peptide exerted strong candidacidal activity *in vitro* and was neutralized by laminarin, suggesting that candidacidal activity was mediated by the interactions between killer peptides and β-glucan moiety present on *Candida* cell surface [158]. Torosantucci et al. prepared a glyco-conjugate vaccine by attaching laminarin with diphtheria toxoid (CRM197), and vaccination with this glyco-conjugate vaccine induced a protective antibody-mediated response in a murine model of disseminated candidiasis. Passive transfer of immune serum (IgG fraction) induced protection in naïve mice and a laminarin binding monoclonal antibody against β-glucan (2G8), was found to confer passive protection against systemic candidiasis infection. Anti-β-glucan antibodies were observed to preferentially bind hyphae of *C. albicans* and could inhibit their growth *in vitro* in the absence of host immune cells. Their results showed that protection mechanisms of the glyco-conjugate vaccine included direct antifungal properties of anti-β-glucan antibodies [146]. Torosantucci et al. also studied the mechanisms of protection induced by anti-β-glucan antibodies by using two laminarin recognizing monoclonal antibodies, 2G8 (IgG2b) and 1E12 (IgM). They found that the IgG2b antibody could recognize β-glucan epitopes present on the fungal cell wall and could confer significant protection against mucosal and systemic candidiasis after passive immunization in rodents. Inhibition of fungal adherence and hyphal growth were the mechanisms of protection by anti-β-1,3-glucan antibodies [148]. It has been reported that healthy humans contain low levels of anti-laminarin antibodies, as compared to other anti-β-glucan and anti-mannan antibodies. Since antifungal efficacy of glyco-conjugate vaccine was tested by the generation of protective anti-laminarin antibodies, Chiani et al. speculated that protective antifungal vaccination in humans should attempt to tip the balance of antifungal antibodies in favor of anti-laminarin antibodies [155]. Adamo et al. evaluated the effect of β- (1,6) branch on the antigenicity of linear β-(1,3) glucans by synthesizing a linear β-(1,3) glucan hexasaccharide, conjugated to CRM197. Immunogenicity analysis showed that the β-(1,3) glucan hexasaccharide-CRM197 conjugate elicited a more homogeneous antibody response with significantly higher IgG titers than the Lam-CRM197 vaccine in mice [175]. Laminarin is a β-glucan ligand of Dectin-1, a pattern-recognition receptor expressed on monocytes, macrophages, and dendritic cells. When laminarin was incorporated into a β-mannan tetanus toxoid conjugate providing a tricomponent conjugate vaccine, immunization of mice with this tricomponent vaccine resulted in improved immune response manifested by high titers of antibodies recognizing *C. albicans* β-mannan antigen. The laminarin vaccine could bind to the Dectin-1 receptor, which resulted in amplification and immunomodulation of the immune response, and also altered the distribution of IgG subclasses [165]. When laminarin was conjugated with a prokaryotically-expressed recombinant calreticulin fragment (CRT), the resulting conjugate exhibited great adjuvanticity and immunogenicity and was found to be capable of eliciting anti-β-glucan IgG (mostly IgG1) responses in not only BALB/c mice but also in athymic nude mice. LAM-CRT was found to activate B-cells via both B-cell receptors and CRT-binding proteins in a synergistic manner, thereby inducing IgG production in the absence of T-cell help [176].

### 2.4. Humoral Immune Responses to Hsp90

Heat shock proteins (Hsps) control basic physiological activities or virulence via interaction with a variety of diverse regulators of cellular signaling pathways. They are expressed in response to thermal stability, morphogenesis, cell cycle regulation, apoptosis, and drug resistance in *Candida* species. Hsps can protect humans from systemic candidiasis and are a major target for the immune system in invasive fungal infections. Particularly, Hsp90 confers protection against *C. albicans* and can bridge the gap between innate and specific humoral immunity. Matthews et al. demonstrated that sera from patients recovering from *C. albicans* mediated systemic infection produced antibodies specific to both *Candida* and heat-shock protein Hsp90 [177]. Moreover, mice vaccinated with serum containing Hsp90-specific antibodies showed increased survival during systemic candidiasis than those receiving normal human serum [177]. Burnie et al. found that a murine monoclonal antibody to conserved Hsp90 epitope (LKVIRK) was protective against *C. albicans* in invasive candidiasis [178]. A human-recombinant antibody against the same epitope was assessed in acute and chronic models of murine invasive candidiasis, and significant renal clearance of *C. albicans* infection and improvement of survival rates in mice suggested that antibody to Hsp90 epitopes could be protective in murine invasive candidiasis [179,180]. Preclinical studies performed using a human-recombinant monoclonal antibody against Hsp90 (Mycograb), found that Mycograb combined with amphotericin-B showed a synergistic protective effect against Hsp90 epitope (NKILKVIRKNIVKK) and imparted protective immunity against *C. albicans*, *C. glabrata,* and *C. krusei* infections [181]. Using a double-blind randomized study, Pachl et al. reported that Mycograb plus lipid-associated amphotericin B produced significant clinical and culture-confirmed improvement in outcome for patients with invasive candidiasis [182]. Mycograb, also known as Efungumab, exerts antifungal activity by inhibiting Hsp90. Clinical data support the use of efungumab in reducing invasive candidiasis in combination with other antifungal agents [183]. Interestingly, Louie et al. found that a variant of Mycograb offered no benefit when combined with amphotericin B in a neutropenic mouse model of systemic candidiasis [184]. The synergistic effect of the Mycograb C28Y variant in the potentiation of amphotericin-B therapy was found non-specific due to the absence of both *in vitro* as well as *in vivo* efficacy in a murine candidiasis model [185,186]. The importance of specific humoral immunity in Hsp90 mediated protection was further validated in a study by Raska *et al*., wherein intradermal administration of Hsp90 protein and DNA vaccine resulted in improved survival rates in BALB/c mice during systemic candidiasis, which correlated with increased levels of anti-*Candida* Hsp90 serum IgG antibodies [187]. Additionally, vaccination of C57BL/6J mice with phage-displaying epitope of *C. albicans* Hsp90 protein elicited protective immune responses against systemic candidiasis through both antibody-mediated and cell-mediated immune responses [188]. Furthermore, vaccination with hybrid-phage particles displaying Hsp90 epitope, induced significant levels of specific antibodies and reduced renal *C. albicans* infection in C57BL/6J mice, confirming that Hsp90 can serve as a potential vaccine candidate for systemic candidiasis by inducing both protective humoral and cellular immunity [189]. Raska et al. further demonstrated that both systemic and mucosal immunization with *Candida* Hsp90 could elicit increased levels of both serum and vaginal Hsp90-specific IgG and IgA antibodies, resulting in enhanced humoral response during experimental vaginal candidiasis [190]. On incorporating His-tagged-recombinant Hsp90 protein into the surface of nickel-chelating liposomes and using muramyl dipeptides as adjuvants, intradermal vaccination of BALB/c mice with this experimental vaccine was seen to induce comparable Th1 and Th2 response, representing a biocompatible platform for the preparation of a recombinant vaccine against candidiasis [191]. Knotigova et al. further evaluated the efficacy of this vaccine with two different adjuvants and observed stimulation of both innate and adaptive immune responses against the nano-formulation of rHsp90 protein [192]. Yang et al. reported that using an adjuvant obtained from the root of *Astragalus membranaceus*, the efficacy of recombinant Hsp90 protein vaccine was enhanced against systemic candidiasis by significant enhancement of IgG antibody titers and various interleukins in the serum of Hsp90-immunized mice [193]. Recently, chitosan hydrogel has been used as an adjuvant, which conferred long-lasting IgG antibody and enhanced Th1, Th2, and Th17 immune responses against a recombinant Hsp90 protein mediated protective immune responses against systemic candidiasis [194].

### 2.5. Humoral Immune Responses to Agglutinin-Like Sequence 3 (Als3)

The Als3 encoded protein, which is a member of the agglutinin-like sequence family, is essentially an agglutinin, playing a crucial role in both fungal adhesion [195] and invasion [196]. It is responsible for binding and attachment of the fungus to diverse host surfaces and also induces endocytosis [196]. Als3 was also found on hyphae isolated from a murine model of disseminated candidiasis [197]. Als3 has been shown to be an invasin that can bind to cadherin proteins, which induce the endocytosis of pathogen by host cells, thereby making it an important virulence factor [197]. However, in a murine model of haematogenously-disseminated candidiasis, a mutant Als3 strain remained as virulent as the wild-type parent strain [198]. The role of Als3 in epithelial adhesion, cell damage, cytokine production, and activation of map-kinase-based signaling pathways has been demonstrated using the mutant Als3 *Candida albicans* strain [199]. A separate study has shown that by helping the secretion of cytokines and chemokines upon *Candida* infection, Als3 protein contributes to *in vitro* fungal killing by oral and vaginal epithelial cells [200]. 

In a seminal study, Spellberg et al. reported that the murine systemic candidiasis model can be used in the evaluation of pathogenesis and antifungal drug efficacy [201]. Using this model, subcutaneous immunization of Als1 protein was seen to increase mice survival during systemic *C. albicans* infection. Moreover, Als1 vaccinated B-cell-deficient mice were not susceptible to intravenous *C. albicans* infection [202,203]. Ibrahim et al. demonstrated that Als1 protein vaccination mediated protection in outbred mice from disseminated candidiasis and also against other virulent strains of *C. albicans* and non-albicans *Candida* species by reducing kidney fungal burden [41]. Spellberg et al. found that, although Als3 immunization was equally effective as Als1 against disseminated candidiasis, it was highly effective than Als1 against both oropharyngeal and vaginal candidiasis, and induced a broader antibody response than the recombinant Als1 protein [204]. However, antibody titers did not correlate with protection against disseminated candidiasis in both Als1 and Als3 vaccinated mice [202,204]. Mice vaccinated with a formulation of Als3 and alum as an adjuvant, exhibited significantly improved survival during systemic *C. albicans* infection [205]. Apart from *Candida* protection, Spellberg et al. reported that Als3 vaccine-induced Th1 immunity was able to generate cross-vaccine protection against *Staphylococcus aureus* mediated bacterial systemic infection [206,207]. Neither adoptive transfer of B lymphocytes from Als3 vaccinated mice nor passive immunization with serum from vaccinated mice conferred protection against systemic *Candida* and *Staphylococcus aureus* infection [206]. Lin et al. demonstrated that the elevated antibody titers induced by Als3 immunization in mice were primarily of IgG and IgG2a subclass [207]. Additionally, the Als3 vaccine formulation could protect mice against both *C. albicans* and *S. aureus* infection by inducing Th1/Th17 immune responses and by enhancing neutrophil phagocytic killing of both organisms [40]. Spellberg et al. reported that, although Als3 immunization could induce primary B-cell responses, producing higher IgG and IgG2a antibody titers, protection against a *Candida* infection was mainly accomplished by the generation of cell-mediated immunity; indicating that antibody response can be used as surrogate markers of vaccine-mediated protection [208]. On analyzing the immunological reactivity of blood from human samples to the rAls3 vaccine, it was seen that healthy individuals have detectable anti-Als3 IgG antibodies in their serum [209]. Uppuluri et al. demonstrated that anti-Als3 antibodies have the potential to disrupt various properties of *C. albicans* like adherence, filamentation, and biofilm formation [28]. A monoclonal antibody C7 was seen to bind to Als3, which inhibited the adhesion of fungus to epithelial surfaces, interfered with fungal filamentation and exhibited fungicidal activity [210]. Further, an Als3-specific monoclonal antibody (3D9.3) was observed to significantly decrease the adhesion of *C. albicans* germ tubes to human epithelial and vascular endothelial cells [211]. 

In a Phase I clinical trial, Schmidt et al. were the first to report that NDV-3 vaccine formulation (using Als3) induced a safe and robust immune response in healthy humans, characterized by significantly high titers of anti-Als3 IgG and IgA1 antibodies [212]. Unlike the limited role reported for B lymphocytes in disseminated systemic candidiasis, NDV-3 vaccination induced protection in mice through both B and T-cell mediated immune response against vulvovaginal candidiasis in B-cell and T-cell deficient mice [213]. However, passive transfer of anti-Als3-N antibodies to naïve mice did not protect against vaginal *Candida* infection [213]. Regardless of the NDV3 dose, both intramuscular and subcutaneous vaccination induced higher anti-Als3 IgG antibody titers *in vivo* [213]. In addition, Yeaman et al. demonstrated that the Als3 vaccine could protect mice from both *Candida* and methicillin-resistant *S. aureus* infections by eliciting a strong B and T-cell response [214]. Furthermore, an Als3 with alum formulation (NDV-3A) was found safe and immunogenic in a clinical trial of patients who had a history of recurrent vulvovaginal candidiasis [215]. NDV-3A vaccination was seen to confer protection against vaginal infections by generating rapid and robust B- and T-cell immune responses [215]. It has been demonstrated that NDV-3A vaccination could protect mice from *C. albicans* infections by inducing higher anti-Als3 antibody titers, which interfered with *C. albicans* ability to adhere and invade endothelial cells and form biofilms *in vitro* [216]. Recently, NDV-3A vaccination was seen to induce protective cross-reactive antibodies and CD4^+^ T-cells against multidrug resistant *C. auris* infection in mice [217].

### 2.6. Humoral Immune Responses to Secreted Aspartyl Proteinase 2 (Sap2)

Sap2 gene belongs to the secreted aspartyl proteinase (Sap) family, which includes 10 members (Sap1-Sap10) [218]. Sap2 is one of the leading vaccine candidates identified from *C. albicans*, and has a well-established role in fungal virulence. The Sap2 genes of *Candida* contributes immensely to fungal pathogenesis by degrading most host proteins at epithelial sites, and can also hydrolyze complement [218]. Gene disruption studies have established the role of various Sap enzymes (including Sap2) in the pathogenicity of *C. albicans,* wherein Sap mutation resulted in attenuation of virulence during disseminated infections [219]. Using triple mutant of Sap gene (Sap4-6), another study confirmed the role of Sap genes in pathogenicity during murine systemic candidiasis [220]. De Bernardis et al. demonstrated that among Sap1 to Sap6, *Candida* mutant strains lacking Sap2 exhibited attenuated virulence in a rat model of vaginal candidiasis [221].

Previous studies have demonstrated that the *C. albicans* proteinases induced antibody responses in humans in response to *Candida* infection [222,223]. Cassone et al. were the first to demonstrate a protective effect of anti-Sap2 antibodies against *C. albicans* vaginal infection in a rat model [110]. De Bernardis et al. reported that immunization with Sap2 antigen or anti-Sap2 monoclonal antibody or anti-Sap2 antibody from vaginal fluids conferred protection in rats against *Candida*-mediated vaginitis [111]. It has been previously-reported that the Sap2 antibody induces a T-cell-dependent protective immune response, which was conferred by specific anti-Sap2 antibodies, which was confirmed by pre-absorption of the fluids with Sap2 that reduced the level of protection [111,224]. In addition to this, protective Sap2-specific antibodies cross-reacted with the other Sap proteins [225,226]. Total IgA, IgG and IgM anti-Sap antibodies were found higher in saliva and serum from HIV (human immunodeficiency virus) infected patients compared to controls [227]. In a longitudinal study by Millon et al., administration of anti-Sap2 antibodies was found to be protective in rats against *C. albicans* mediated vulvovaginal candidiasis [228]. Ghadjari et al. obtained sera from patients who had both oral and systemic candidiasis, and identified six Sap2-specific IgG and IgM B-cell epitopes, which may play a role in conferring protection against disseminated candidiasis [229].

Animals infected with *C. albicans* had increased levels of anti-mannan and anti-Sap2 antibodies in vaginal fluid, which mediated protection against vaginal *C. albicans* infection [122]. Both intranasal and intravaginal administration of Sap2 along with cholera toxin induced specific antibody response, which was found to be protective against *C. albicans* mediated vaginitis in rats [230]. Further, in atopic dermatitis, Suenobu et al. showed that nasal vaccination with Sap2 along with cholera toxin induced an anti-Sap2 IgA antibody response, which helps in eliminating *C. albicans* from the gastrointestinal tract of dermatitis patients [231]. Vilanova et al. were the first to report that immunization with recombinant Sap2 protein conferred protection against systemic candidiasis in mice and passive transfer of anti-Sap2 antibody (IgG) significantly lowered the fungal burden in kidneys during *C. albicans* infection, establishing the protective effect of anti-Sap2 antibodies against *C. albicans* mediated disseminated *Candida* infection [232]. In a mucosal model, mice immunized intranasally with Sap2 exhibited reduced fungal burdens after both oral and vaginal challenge with *C. albicans* [233].

The antibodies generated against Sap2 immunization have a role in enzyme neutralization, and the hypothesis that *Candida* infections can be attenuated by Sap2 inhibition has been supported to some extent in animal models by treatment with aspartic protease inhibitor pepstatin in mucosal and peritoneal *Candida* infections [234]. Using a rat model of vaginal candidiasis, another study demonstrated successful protection against *C. albicans* vaginitis, through induction of increased anti-Sap2 IgG and IgA antibodies titers in Sap2 immunized rats [235]. Furthermore, protection conferred by passive transfer of immune vaginal fluid and anti-rSap2 IgM and IgG monoclonal antibodies confirmed the protective effects of Sap2-specific antibodies [235].

Bernardis et al. demonstrated that intramuscular immunization with a virosomal formulation of the Sap2 vaccine (PEV-7) induces protective antibody response in mouse and rat models [236]. Immunization with recombinant Sap2 protein by intravaginal or intramuscular routes generated anti-Sap2 antibodies (IgG and IgA) in rat vaginal fluid and rats immunized via intravaginal route with PEV7 exhibited antibody-mediated protection against *C. albicans* vaginitis [236]. The PEV-7 virosomal vaccine formulation has successfully completed a Phase I clinical trial [237] and PEV7 vaccination either via intramuscular injections or by intravaginal capsules was observed to induce a strong B-cell mediated immune response in vaginal and cervical samples [104]. A study showed that BALB/c mice vaccinated with either hybrid phage displaying Sap2 epitope SLAQVKYTSASSI or recombinant Sap2 protein induced strong cellular and protective humoral responses against *C. albicans* infection [238]. Notably, a separate study found that, although Sap2 immunization was protective against systemic *C. albicans* infection in mice, immunization with murine DCs sensitized by pulsing with Sap2 protein was more immunoprotective [239]. Pericolini et al. showed that vaccine-induced or passively administered anti-Sap2 antibodies contributed to protection against *Candida* vaginitis by inhibiting the inflammatory response during vaginitis caused by *C. albicans* in mice [240]. Furthermore, anti-Sap2 antibodies selected from a human Fab antibody library provided protection to control vaginitis [240]. More recently, it has been reported that both virus nanofibers displaying Sap2 epitope and recombinant Sap2 vaccination could protect mice by inducing humoral and cell-mediated immune response against *C. albicans* infection [241]. Additionally, mice treated with anti-rSap2 single chain variable fragments exhibited significantly increased survival rates and had significantly decreased fungal burdens compared to control groups in a murine model of *C. albicans* mediated systemic infection [242]. A recent report has shown that Sap2 vaccination resulted in increased titers of anti-Sap2 antibodies, which could bind the whole fungus [29]. Anti-Sap2 antibodies exhibited increased *Candida* biofilm inhibition ability *in vitro*, enhanced neutrophil-mediated fungal killing, and protected naïve mice against systemic infection on passive transfer. Additionally, the findings of this study suggested a role of *Candida*-specific B1 and B2 B-cells during early stages of Sap2-mediated immune response [29].

### 2.7. Humoral Immune Responses to Hyphally Regulated Protein 1 (Hyr1)

HYR1 is a hyphae-expressed gene, required for hyphal growth and virulence. Bailey et al. showed that Hyr1 protein was strictly expressed on *C. albicans* hyphae, and has no effect on the fungus germination [243]. Hyr1 is known to contribute to *C. albicans* virulence by resisting phagocyte killing, a major host defense mechanism against candidiasis. Luo G et al. reported that rHyr1 protein plus alum adjuvant vaccination enhanced survival and reduced fungal burden in both immunocompetent and immunocompromised mice against disseminated candidiasis [244]. Additionally, passive immunization with anti-Hyr1protein polyclonal antibodies protected mice against *C. albicans* infection by directly neutralizing Hyr1 protein *in vitro*, resulting in enhanced mouse neutrophil-killing activity [244]. Both active and passive immunization with the rHyr1p-N (a recombinant N-terminal fragment of *C. albicans* Hyr1 protein), has been shown to protect mice against lethal candidemia [245]. It has been suggested that vaccination with Hyr1 is protective against systemic candidiasis most likely due to a direct, non-opsonic enhancement of neutrophil killing by anti-Hyr1 antibodies [149]. Uppuluri et al. reported that the active vaccination with rHyr1p or passive immunization with anti-Hyr1p antibodies protected mice from *Acinetobacter* *baumannii* mediated bacteremia and pneumonia [246]. Polyclonal antibodies raised against peptides derived from the Hyr1p-N were seen to inhibit *C. albicans* and *A. baumannii* mixed biofilm formation *in vitro* [246], and monoclonal IgM antibodies targeting *C. albicans* Hyr1 protein provided cross-kingdom protection against gram-negative bacteria [247]. Rudkin et al. isolated single class switched memory B-cells from donors serum-positive for anti-*Candida* IgG, which were differentiated *in vitro* and screened against recombinant Hyr1 cell wall protein and whole fungal cell wall preparations [90]. The Hyr1-specific single human B-cell-derived monoclonal anti-*Candida* antibodies enhanced phagocytosis and protected against disseminated candidiasis [90]. These studies support that Hyr1 vaccination approach is based on generation of neutralizing and/or protective antibodies.

### 2.8. Humoral Immune Responses to Hyphal Wall Protein 1 (Hwp1)

The Hwp1 gene encodes a *C. albicans* hyphal cell wall protein, which is a substrate for mammalian transglutaminases. Hwp1 is an adhesion protein, expressed on the hyphal cell wall of *C. albicans*, which promotes the cross-linking of fungus with epithelial cells. It is a member of the GPI (glycosylphosphatidylinositol) anchor-dependent family of cell wall proteins, which is known to participate in covalent bonds formation with primary amines and buccal epithelial cells. Hwp1 protein has been shown to be important for both *in vivo* hyphal development and pathogenicity of *C. albicans* [248]. Expression of Hwp1 was shown to be critically required for biofilm formation in both *in vitro* and *in vivo* studies, making it a potential therapeutic target [249]. Studies have shown that Hwp1 expression is dependent on the transcription factor Bcr1, a zinc finger protein [250]. Naglik et al. reported the presence of systemic adaptive antibody responses to Hwp1 (by testing Hwp1-specific IgG and IgA titers) in candidiasis patients and healthy adults, thereby indicating a consistent role of Hwp1 in the pathogenesis of candidiasis [251]. Interestingly, humoral immunity has been observed to link *C. albicans* infection and celiac disease, wherein patients with celiac disease had high levels of anti-Hwp1 antibodies [252]. Vaccination with glycopeptide conjugate consisting of β-mannan polysaccharide combined with Hwp1 peptide epitopes, showed protection against experimental disseminated candidiasis in mice, by favoring production of protective and specific antibodies [132]. Recently, Rosario-Colon et al. found that *Candida* Hwp1-specific monoclonal antibodies could protect mice against invasive *C. auris* infection, by significantly enhancing survival and reducing fungal burdens [253].

### 2.9. Humoral Immune Responses to Enolase (Eno)

Enolase is an abundantly expressed cytosolic enzyme involved in the glycolysis pathway. It is secreted in the extracellular medium and is also found on the surface of fungal cell wall [254]. It mediates fungal adhesion to human tissues, activation of fibrinolysis and extracellular matrix degradation [254,255]. It has been shown that an anti-enolase antibody interfered with *C. albicans* adhesion with epithelial cells [254]. The predominant antibody responses to enolase in multiple studies suggests that antibody generation to enolase is an early and sensitive indicator of proliferation of *C. albicans* in mice. In humans, antibody responses to enolase are low or absent during colonization but increase after disseminated infection in immunocompetent hosts. In a study by Sundstorm et al., enolase was found to be an immunodominant humoral immunogen, which induced lymphocyte activation and stimulated both cell-mediated and humoral immune responses in mice [256]. Passive transfer of anti-enolase antibodies was found to be partially protective against systemic candidiasis in mice [257]. In a separate study, mice immunized with enolase plus IL-12 showed increased antibody titres against enolase, as well as increased median survival time and decreased fungal burden in kidneys, in comparison to non-immunized or IL-12-treated mice [258]. Since increased survival was also observed in B-cell deficient mice, enolase mediated protection was attributed due to cell-mediated immunity of predominant Th1 type, despite the high immunogenicity observed for recombinant enolase [258]. Another study showed that mice vaccinated with recombinant enolase protein were effectively protected against disseminated *C. albicans* infection. Passive transfer of enolase-specific antiserum reduced fungal burden in the target organs, and enolase-specific IgG1 and IgG2a antibodies could enhance neutrophil-mediated killing of *C. albicans* strains by opsonization [259]. It has been suggested that serological detection of IgG antibodies against *Candida* enolase and fructose-bisphosphate aldolase can be used in the diagnosis of systemic candidiasis [260,261]. Shibasaki et al. described that oral administration of *Saccharomyces cerevisiae* cells displaying enolase 1 antigen on their surfaces could elicit an immune response and aid the survival of mice challenged with *C. albicans* [262]. Another study by He et al. analyzed the serological response to recombinant proteins including enolase (rEno1), phosphoglycerate kinase (rPgk1), and β-glucosidase (rBgl2) in a murine model of systemic candidiasis, in which, rEno1 showed strong serological response than rPgk1 and rBgl2 [263]. Recently, Leu et al. isolated a single-chain variable fragment monoclonal antibody (CaS1) against recombinant *C. albicans* enolase by phage display technology and evaluated its role during *in vitro* and *in vivo* conditions. This monoclonal antibody inhibited the growth as well as plasminogen binding activity of *C. albicans*. Further, CaS1 administration enhanced survival time, reduced fungal burdens and also reduced the levels of inflammatory cytokines in a mice model of *in vivo* candidiasis [264].

### 2.10. Humoral Immune Responses to Phospholipase B (PLB)

Phospholipases are a heterogeneous group of enzymes that hydrolyze one or more ester linkages in glycerophospholipids, and have been proposed to contribute to the virulence of *C. albicans*. Phospholipase B (PLB) enzyme has both hydrolase and acyltransferase activities, and can be found in secreted or intracellular forms [265]. It plays a central role in cellular processes such as signal transduction and inflammation through its effect on the metabolism of phospholipids and lysophospholipids. Theiss et al. demonstrated that the PLB5 gene inactivation in *C. albicans* reduced the activity of phospholipase A2 enzyme and also attenuated fungal virulence [266]. The confirmation of PLB as a virulence factor using animal models of disseminated candidiasis; coupled with its detection in other pathogenic fungi, makes it a potential therapeutic target [267]. Using scanning electron microscopy, Leidich et al. showed significantly lower penetration of PLB-deficient *Candida* strain than the parent *Candida* strain to both HUVEC and HT-29 epithelial cells *in vitro* [268]. These studies indicate that *Candida* PLB may play a crucial role in the dissemination of *C. albicans* via gastrointestinal and hematogenous routes [267,268]. Mukherjee et al. demonstrated that reintroduction of PLB1 gene into *C. albicans* could restore fungal virulence *in vivo*. The study used anti-PLB1 antibody to show that PLB1 is secreted during the invasion of the gastric mucosa by both parental and revertant strains, suggesting for the use of phospholipases as a vaccine candidate [269]. Studies have shown that serum of systemic candidiasis patients contain antibodies reacted with purified *Candida* PLB, making it an attractive candidate for diagnostic use as well. Not much is reported in literature regarding B-cell or antibody mediated responses against PLB, except that it can be used as a potential vaccine candidate due to its hyphal-specific nature [104,270].

### 2.11. Humoral Immune Responses to Fructose-Bisphosphate Aldolase (Fba1)

Fructose-bisphosphate aldolase (Fba1) is a multifunctional protein and an important enzyme of glycolytic pathway. It can facilitate fungal attachment to human cells or abiotic surfaces, and protects *Candida* cells from the host’s immune system [271]. In addition to this, it promotes the detoxification of reactive oxygen species generated during respiratory burst. Proteomics analysis revealed that Fba1 is the most abundant and stable enzyme in *Candida* and is considered to be one of the main immunodominant proteins [271]. When β-mannan was conjugated to Fba peptide, vaccination with the Fba peptide was observed to induce protective antibody production and protection against disseminated candidiasis in mice [132]. Passive transfer of immune sera from Fba-vaccinated mice conferred protection in naïve mice. Additionally, an IgM monoclonal antibody specific for Fba peptide, protected mice against disseminated candidiasis, providing strong evidence that anti-Fba antibodies contribute to protection [103]. Using a 14-mer peptide from Fba for generating a self-adjuvanting vaccine, Xin et al. demonstrated that the conjugate induced protective antibody responses against both the glycan and peptide parts of the vaccine, which was confirmed by passive transfer of protective sera [133]. Not only did Fba peptide-pulsed dendritic cell vaccination induce a high degree of protection against disseminated candidiasis in immunocompetent mice, both active immunizations with Fba peptide-DC vaccines and passive transfer with antibodies protected neutropenic mice against disseminated candidiasis [272]. Phage vaccines displaying YGKDVKDLFDYAQE epitope from Fba1 protein was observed to confer protection against systemic candidiasis in a mouse model, mainly by inducing humoral and cellular immune responses. The vaccine reduced the fungal burden and relieved kidney damage in infected mice, thereby improving their survival rates significantly [273]. A study by Medrano-Diaz et al. found that recombinant Fba immunization confers immunity in mice against systemic *C. glabrata* infection and has an immunodominant role [274]. Furthermore, immunization with three mimotope-peptide conjugate vaccines was also able to induce specific antibody responses, and protected against disseminated candidiasis in mice. Antibody-mediated protection has been demonstrated for Fba1 using passive transfer studies, and complement activation along with interference with hyphal growth have been identified as protection mechanisms [275]. Adams et al. demonstrated that vaccination with an Fba-methionine synthase peptide construct (MP12), protected mice against disseminated candidiasis and was seen to induce IgG1 and IgG2a antibodies, indicating a role of humoral immunity in protection [276]. Using an immuno-informatics approach, Elhasan et al. predicted the most conserved and immunogenic B- and T-cell epitopes from Fba1 protein of *C. glabrata*, for designing an effective epitope-based peptide vaccine [271].

### 2.12. Humoral Immune Responses to Pyruvate Kinase (Pk)

The *C. albicans* cell wall protein Pyruvate kinase (Pk), which is a glycolytic enzyme, is of special interest, due to its involvement in oxidative stress as well as in the formation of biofilms. On screening for *C. albicans* sequences that encode proteins that are immunogenic during infections in humans, Swoboda et al. identified few glycolytic enzymes, including pyruvate kinase and alcohol dehydrogenase as non-ubiquitous immunogens during *C. albicans* infections [277]. Using a proteomics approach, Fernandez-Arena et al. identified pyruvate kinase as one of the antigens that induced protective IgG2a antibody isotype in the sera of vaccinated animals [278]. On assaying *C. albicans* infected mice immune sera obtained on different days post-infection using two-dimensional polyacrylamide gel electrophoresis, Pitarch et al. demonstrated the presence of pyruvate kinase as an immunoreactive antigen [279]. More recently, Medrano-Diaz et al. reported that vaccination with recombinant Pk antigen could confer protection against systemic *C. albicans* infection in mice and displayed the highest immunogenicity [274].

### 2.13. Humoral Immune Responses to Superoxide Dismutase (Sod5)

Superoxide dismutase (Sod) are antioxidant enzymes that convert superoxide radicals into less damaging hydrogen peroxide. In *C. albicans*, six members of the SOD gene family have been identified, amongst which, Sod5 is exclusively extracellular, and is attached to the fungal cell surface via GPI anchors [280]. Sod5 is upregulated under osmotic and oxidative stress conditions, as well as during yeast-to-hyphae transition [281] and is required for virulence of *C. albicans* in invasive mouse models [282]. Since Sod5 mainly exists as a hyphally-associated cell wall protein [283], it is considered to be a promising vaccine candidate against systemic candidiasis [104,270]. Using a tail vein injection model for *C. albicans* mediated disseminated candidiasis, it has been seen that the loss of the Sod5 gene resulted in attenuation of fungal virulence [281]. *C. albicans* mutants lacking Sod5 were more susceptible to killing by macrophages and neutrophils and were associated with increased reactive oxygen species production by macrophages and neutrophils [284]. Additionally, Robinett et al. demonstrated that deletion of the Sod5 gene could eliminate *Candida* biofilm formation on venous catheters in a rodent model [285]. It has been speculated that inhibiting or blocking the extracellular Sod enzymes of *C. albicans* may be a novel therapeutic approach to combat systemic fungal disease [284].

### 2.14. Humoral Immune Responses to Malate Dehydrogenase (Mdh1)

*C. albicans* malate dehydrogenase (Mdh1) has been screened by few proteome studies as a candidate for a vaccine against candidiasis [278,286]. The Mdh1 protein is essential for completing the TCA (tricarboxylic acid cycle) cycle and is involved in the aerobic generation of energy through participation in the malate-aspartate shuttle. It is regarded as a candidate vaccine antigen against candidiasis because it was detected at all time points studied without large variations in its relative abundance [287]. On evaluating recombinant Mdh1 protein as an antigenic candidate for a vaccine against candidiasis, Shibasaki et al. found that both subcutaneous and intradermal administration of recombinant Mdh1 protein induced significantly higher antibody responses and elicited significant protection against *C. albicans* mediated systemic infection in mice [288].

## 3. Conclusions

In the past few decades, significant progress has been made in understanding the protective host immune responses generated against invasive *Candida* infections. The fungal pathogen contains certain virulence factors on its cell wall, which contributes to immune evasion. Generation of innate and adaptive immune responses are essential for fungal clearance and the major players of innate and adaptive immunity include phagocytes, cytokines, complement proteins, T-cells, B-cells, and antibodies. Although cellular immune responses are critical for conferring protection against systemic fungal infection, the potential of humoral immunity in contributing to protective responses is still debated. In various studies, several *Candida* virulence antigens, like cell wall polysaccharides, surface proteins, and enzymes have been investigated as vaccine candidates to assess the protective immune responses against fungal challenge in animal models of systemic candidiasis. Many different subunit and conjugate vaccines have been prepared by using Sap2, Als3, Hyr1, enolase, mannan, and glucan, out of which only Sap2 and Als3 vaccines have successfully completed phase-1 trials while the rest of the vaccines are under preclinical trials. Most antifungal vaccines exert protection by inducing either (or both) B-cell and T-cell responses. In this review, we have consolidated the currently-available information on *Candida* vaccine antigens, induced B-cells, antibody immune responses and latest findings on the role of different types of antibodies in the antifungal defense against *Candida* infections.

Data from multiple literature reports summarized in this review clearly show that antibodies induced in response to leading vaccine candidates do contribute to protection against systemic candidiasis by utilizing a variety of mechanisms (Table 1). Anti-Sap2 antibodies exhibit properties like enzyme neutralization, inhibition of biofilm formation, and enhancement of neutrophil mediated killing. Although Als3 mediated protection is mainly due to T-cell-mediated immune responses, primary B-cell responses produce increased antibody titers, which are used as markers to predict vaccine-mediated protection. While anti-Als3 antibodies are known to inhibit fungal adherence, filamentation, and biofilm formation, anti-Hyr1 antibodies inhibit biofilm formation and have a role in the direct, non-opsonic enhancement of neutrophil mediated fungal killing. Anti-enolase antibodies are known to inhibit fungal attachment on host cells and enhance neutrophil mediated killing of *C. albicans* by opsonization. Antibodies against Hsp90 exhibit direct antifungal activity by inhibiting *Candida* Hsp90 antigen, which is indispensable for fungus viability. Anti-mannan antibodies can activate both classical and alternate pathway of complement system and enhance opsonophagocytic killing of *C. albicans.* Direct antifungal activity is also reported for β-glucan and laminarin antibodies, which are known to inhibit fungal growth and adherence. Comparatively fewer reports are available for identifying mechanisms of protection for antibodies generated against Hwp1, PLB, Pk, Fba, Sod5, and Mdh1 vaccine antigens. In addition to functioning as antigen-presenting cells that contribute to the activation and differentiation of T-cells, B-cells can directly bind to fungal antigens and differentiate into antibody producing plasma cells which are the key players of antifungal humoral immune response. B-cells also produce memory B-cells that prevent secondary infections. B-cell-deficient mice fail to develop Th2-dependent immunity and have decreased levels of IL-4, IL-10, and TGF-β cytokines. Further, an antibody-independent role of B-cells during fungal infection has been seen on depleting B- cells *in vitro*. In addition, B1 B-cells have also been implicated in contributing to innate immunity against *Candida* species.

Multiple studies in the literature provide support in favor of using passive antibody therapy during disseminated candidiasis, especially in immunocompromised hosts and high-risk patients. Although there is strong evidence from animal studies, substantial experimental proof from clinical studies is still lacking. Nevertheless, anti-*Candida* monoclonal antibodies and human-recombinant antibodies are considered promising immunotherapeutics against systemic candidiasis. Monoclonal antibodies generated against leading vaccine antigens have been shown to elicit various anti-*Candida* immune responses such as opsonophagocytosis, neutralization, fungal growth inhibition, biofilm inhibition, and direct candidacidal activity (Table 2).

Likewise, anti-idiotypic antibodies and mimotopes which recognize yeast killer toxins are known to exhibit direct candidacidal activity. Antibody-derived peptides and single chain variable fragments have been used in many studies for active or passive vaccination in mice/rat model of experimental disseminated candidiasis. The finding that anti-*Candida* antibodies against a single determinant (β-glucan) can cross-react and confer protection against different fungal pathogens, makes a strong case for utilizing antibodies in adjunctive therapy. Synergistic combinations of monoclonal antibodies and antifungal drugs would not only provide a more potent and effective solution for combating emerging drug resistance in *Candida* species, but the enhanced specificity is also expected to improve clinical outcomes. Developing novel immunomodulatory approaches using monoclonal antibodies combined with administration of recombinant cytokines is another promising way for enhancing their therapeutic effect. In addition, new and upcoming technologies, such as multivalent antibodies, antibody-drug conjugates or antibody-antibiotic conjugates can be utilized for treatment of life-threatening invasive fungal infections.

## 4. Future Perspective

- The serious threat posed by invasive fungal infections is a persisting problem and new therapeutic options are urgently required including vaccine/s and/or antibody-based therapies. Challenges associated with the extensive use of antifungal drugs like antifungal drug-associated toxicity and emergence of antifungal drug resistance, along with increased incidences of infections due to multi-drug resistant non-albicans *Candida* species merits the need for developing alternate immunotherapies against invasive candidiasis.
- Due to the promising potential of humoral immunity in conferring antifungal protection, identification of fungal antigens which elicit protective antibodies will be crucial for designing effective multi-valent and/or multi-epitope-based anti-*Candida* vaccine/s.
- There is a need to synthesize novel peptides and oligosaccharides using a combination of protective antigens that could be used to make multi-epitope conjugate vaccines including B-cell and T-cell epitopes for generating memory. Although few experimental vaccines have been designed, extensive animal-based studies are lacking.
- Additionally, identification, isolation and characterization of protective anti-*Candida* monoclonal antibodies along with elucidation of their protection mechanisms, will be beneficial in generating more precise antibody-based therapies. More clinical trials are needed to validate passive antibody therapy against invasive candidiasis.
- Novel cytokine-based adjunctive immunotherapies and immunomodulators should be explored for the treatment of invasive candidiasis. More studies are needed to demonstrate the feasibility of such immunotherapies for improving the prognosis of invasive candidiasis.
- Synergistic use of monoclonal antibodies in combination with anti-fungal drugs has been seen to impart cooperative protection against *Candida*. More studies are needed to assess the safety and efficacy of combination therapies.
- Susceptibility of immunocompromised individuals having deficiencies in an immune cell repertoire to invasive candidiasis justifies future research for characterization of the protective B-cell and T-cell repertoires, aiding the development of novel vaccination strategies.

## Figures and Tables

**Table 1:** Protective immune responses generated by experimental vaccines against *Candida* antigens.

S.N	*Candida* Antigen	Experimental Vaccine	Implicated Humoral Mechanisms of Protection	References
1.	Mannan	liposome-encapsulated mannan	agglutination of *Candida* cells, *in vitro* neutrophil candidacidal activity, complement aided protection	[100,113,138]
		mannan extracts	anti-adhesion or anti-germ tube formation effects	[110]
		mannan–BSA conjugate	antibody isotype switching	[117,138]
		mannan-HSA conjugate	inhibition of *C. albicans* growth, B-cell immune-enhancement and antifungal activity	[123,130,131,141]
		mannan oligosaccharide conjugates	complement fixation and complement mediated clearance of *C. albicans*, enhanced candidacidal activity, enhanced phagocytic activity, induction of respiratory burst of granulocytes, Immunomodulation and antibody isotype switching	[121,128,129,134,135,136,137,139,142]
2.	β-glucan	β-glucan-conjugate vaccine	opsonization by enhanced phagocyte mediated extracellular hyphal killing, *in vitro* growth-inhibition of *C. albicans*, inhibition of hyphal growth, invasion and adherence.	[146,148,151,152,175]
		β-glucan oligosaccharides plus keyhole limpet hemocyanin (KLH)	antibody class switching	[167,168]
		linear β-(1→3)-nonaglucoside plus BSA (G9	immunomodulation, candidacidal activity, inhibition of fungal growth adherence and dissemination, enhanced conidial phagocytosis.	[169,170]
		OVA plus Curdlan	B-cell activation and enhancement of antibody response.	[172]
		β-glucan	Induction of fungicidal activity of hemocytes.	[163,164]
3.	Laminarin	Laminarin-CRM197 conjugate	enhanced opsonisation and phagocyte mediated hyphal killing, *in vitro* growth-inhibition of *C. albicans*, inhibition of hyphal growth, fungal invasion and adherence, biofilm inhibition, direct antifungal properties.	[146,148,152,154,175]
		Laminarin conjugated with recombinant calreticulin fragment (LAM-CRT conjugate)	Inhibition of *C. albicans* growth *in vitro*.	[177]
		β-mannan trisaccharide-(tetanus toxoid)-Laminarin tricomponent conjugate	immunomodulation of the immune response, isotype switching of IgG subclasses.	[165]
4.	Hsp90	recombinant Hsp90 protein	antibody mediated neutralization	[180,187,190,193,194]
		Hybrid-phage displaying Hsp90 epitope	neutralization	[188,189]
		proteoliposomal formulation of Hsp90	immunomodulation and neutralization	[191,192]
		Hsp90 expressing DNA vaccine	neutralization	[187]
5.	Als3	Recombinant Als3 protein	enhanced phagocyte recruitment and inflammatory cytokine production	[40,204,205,206,207,208,209]
		Als3 with alum formulation (NDV-3A)	recruitment of phagocytes, enhanced neutrophil-mediated killing of *C. albicans*, enhanced opsonophagocytic killing by macrophages, interference with fungal adherence and invasion, inhibition of biofilm formation, reduction in hyphal elongation, inhibition of yeast dispersal from hyphal layers of biofilms.	[28,212,213,214,215,216,217]
6.	Sap2	recombinant Sap2 protein	Sap2-specific antibodies induced enzyme neutralization, inhibited inflammatory response, exhibited increased *Candida* biofilm inhibition ability *in vitro*, and enhanced neutrophil-mediated fungal killing	[29,111,230,231,232,233,235,238,239,240,241]
		virosomal formulation of Sap2 (PEV-7)	Sap2 neutralization, inhibition of fungal adherence.	[236,237]
		hybrid phage displaying Sap2 epitope SLAQVKYTSASSI	Induction of Sap2 specific antibody production, immunomodulation	[238]
		epitope peptide of Sap2 displayed on virus nanofibers	Sap2 antibodies prevented fungal adhesion and colonization of *C. albicans* in the host.	[241]
7.	Hyr1	recombinant Hyr1 protein	opsonophagocytosis, enhanced neutrophil-mediated killing activity, inhibition of biofilm formation.	[90,149,244,245,246]
8.	Hwp1	Hwp1 glycopeptide conjugate	inhibition of biofilm formation, antibody-mediated growth inhibition of *C. albicans*	[103,132]
9.	Enolase	recombinant enolase protein	enolase-specific IgG1 and IgG2a antibodies enhanced neutrophil-mediated killing of *C. albicans,* opsonophagocytosis and Induced complement activation.	[257,258,259,262]
		*Saccharomyces cerevisiae* cells displaying enolase 1	not determined	[262]
		β-(Mannan trisaccharide)-Eno1 peptide conjugate vaccine	not determined	[103,132]
10.	Fba1	recombinant Fba1 protein	not determined	[273,274]
		Fba peptide conjugated with β-1,2-mannotriose	complement activation and interference with *Candida* hyphal growth	[103,132,133]
		Fba peptide pulsed DC formulation	not determined	[272]
		Phage displaying Fba epitope	not determined	[273]
		Fba peptide and mimotope conjugate	not determined	[275]
		Fba-methionine synthase peptide construct (MP12)	phagocytosis, antibody-dependent complement activation	[276]
11.	Pk	recombinant Pk protein	not determined	[274]
12.	Mdh1	recombinant Mdh1p protein	not determined	[288]

**Table 2:** List of protective monoclonal antibodies generated against *Candida* antigens.

S.N.	*Candida* Antigen	Monoclonal Antibodies	Mechanisms of Protection	References
1	Mannan	B6.1 (IgM)	neutrophil mediated ingestion and killing of *Candida*	[108,109]
		C3.1 (IgG)	complement fixation, enhanced phagocytosis and fungal killing	[118,119,120]
		M1g1 (IgG)	complement activation and phagocytic killing of *C. albicans*	[125,126]
		AF1 (IgM)	anticandidal protection in vaginitis	[111]
2.	β-glucan	3G11 (IgG1)	enhanced *Candida* phagocytosis, inhibition of fungal growth	[170]
		5H5 (IgG3)	cooperative *Candida* growth inhibitory activity with fluconazole	[170]
		2G8 (IgG2b)	inhibited *Candida* adherence and exhibited direct fungal growth inhibitory activity	[146,148,149]
		1E12 (IgM)	non-protective	[148]
3.	Hsp90	Mycograb (Efungumab)	exerted antifungal activity by inhibiting Hsp90	[181,182,183]
4.	Als3	C7 (IgM)	inhibition of adherence, interference with fungal filamentation and exhibited direct candidacidal activity	[210]
		3D9.3 (IgM)	decreases adhesion of *C. albicans* germ tubes to epithelial and endothelial cells	[211]
		3-A5 (IgG1)	blocked adhesion of *C. albicans* to endothelial and epithelial cells.	[197,289]
		113 (IgG1	blocked adhesion of *C. albicans* to endothelial and epithelial cells.	[197,289]
5.	Sap2	GF1 (IgG1)	protected against *C. albicans* vaginal challenge	[111]
		NL2/2A8 (IgM)	rapid clearance of fungal cells from mucosal sites	[235]
		NL2/9B9 (IgM)	rapid clearance of fungal cells from mucosal sites	[235]
6.	Hwp1	6H1 (IgG2b)	induced significant protection against *C. auris* infection *in vivo*	[253]
7.	Hyr1	17 human anti-Hyr mAbs (IgG1)	enhanced opsonophagocytosis of *C. albicans* and *C. auris*	[90]
		H3 (IgM)	cross kingdom immune-protection against gram negative bacteria	[247]
		H4 (IgM)	cross kingdom immune-protection against gram negative bacteria	[247]
8.	Enolase	CaS1 (Single chain variable fragment)	inhibited the growth as well as plasminogen binding activity of *C. albicans*	[264]
		12D9 (IgG)	conferred antifungal activity by neutralizing Eno1, enhanced opsonophagocytosis and neutrophil mediated killing	[290]
9.	Fba1	E2-9 (IgM)	complement activation and interference with *Candida* hyphal growth	[103]

## Data Availability

Not applicable.
